# Assessment of levothyroxine therapy adequacy in low-risk differentiated thyroid carcinoma: a multicenter cohort study

**DOI:** 10.3389/fendo.2025.1652862

**Published:** 2025-12-12

**Authors:** Juan J. Díez, Emma Anda, Victoria Alcazar, Juan C. Galofré, Cristina Familiar, María J. Pamplona-Civera, Silvia González-Martínez, Ana Herrero-Ruiz, María R. Alhambra, Alejandra Planas, Cecilia Sánchez-Ragnarsson, Tomás Martín, Beatriz Rodriguez-Jiménez, Mireia Mora, Aida Orois, Reinaldo Sánchez-Barrera, Julia Sastre

**Affiliations:** 1 Department of Endocrinology, Hospital Universitario Puerta de Hierro Majadahonda, Majadahonda, Spain; 2 Instituto de Investigación Sanitaria Puerta de Hierro Segovia de Arana, Majadahonda, Spain; 3 Department of Medicine, Universidad Autónoma de Madrid, Madrid, Spain; 4 Department of Endocrinology, Hospital Universitario de Navarra, Pamplona, Spain; 5 Department of Endocrinology, Hospital Severo Ochoa, Leganés, Spain; 6 Department of Endocrinology, Clínica Universidad de Navarra, Pamplona, Spain; 7 Department of Endocrinology, Hospital Clínico San Carlos, Madrid, Spain; 8 Department of Endocrinology, Hospital Royo Villanova, Zaragoza, Spain; 9 Department of Endocrinology, Hospital Universitario de Cabueñes, Gijón, Spain; 10 Instituto de Investigación Sanitaria del Principado de Asturias (ISPA), Oviedo, Spain; 11 Department of Endocrinology, Hospital Universitario de Salamanca, Salamanca, Spain; 12 Unidad de Gestión Clínica (UGC) Endocrinología y Nutrición, Hospital Universitario Reina Sofía, Córdoba, Spain; 13 Instituto Maimónides de Investigación Biomédica de Córdoba (IMIBIC), Córdoba, Spain; 14 Department of Endocrinology, Vall d’Hebron Hospital Campus, Barcelona, Spain; 15 Department of Endocrinology, Hospital Universitario Central de Asturias, Oviedo, Spain; 16 Department of Endocrinology, Hospital Universitario Virgen Macarena, Sevilla, Spain; 17 Department of Endocrinology, Hospital Clínic, Barcelona, Spain; 18 Department of Endocrinology, Hospital Universitario de Bellvitge, Barcelona, Spain; 19 Department of Endocrinology, Hospital Universitario de Toledo, Toledo, Spain

**Keywords:** differentiated thyroid cancer, levothyroxine, thyrotropin suppression, adequacy, dynamic risk stratification

## Abstract

**Objective:**

This study aimed to evaluate the adequacy of levothyroxine therapy, assessed by serum thyrotropin (TSH) levels, in patients with low-risk differentiated thyroid carcinoma (DTC).

**Methods:**

We conducted a multicenter, retrospective cohort study including patients with low-risk DTC. Dynamic risk stratification was performed 12 months after initial treatment and at the last follow-up visit according to the 2015 American Thyroid Association (ATA) guidelines. Patients were categorized based on treatment response as excellent, indeterminate, biochemical incomplete, and structural incomplete. Levothyroxine adequacy was determined according to ATA-recommended TSH target values.

**Results:**

A total of 1016 patients (median age, 48 years; 80.7% women; 91.4% papillary thyroid carcinoma) were followed for a median of 6.6 years. Total thyroidectomy was performed in 935 (92.0%) (plus radioiodine in 667), while 81 (8.0%) underwent lobectomy. An excellent response was observed in 633 (62.3%) at 12 months and in 761 (77.8%) at the last follow-up. Treatment adequacy increased from 264 (26.0%) at 12 months to 387 (39.5%) at the final visit (P<0.001). Among patients with excellent response, treatment adequacy rose from 25.8% to 44.3% (P<0.001). At the last visit, inadequacy was primarily due to excessive levothyroxine in patients with excellent response (30.5%), and insufficient dosing in those with indeterminate or biochemical incomplete response (62.2% and 50.0%, respectively). Levothyroxine dose instability correlated significantly with treatment inadequacy (P<0.001).

**Conclusion:**

The low rate of treatment adequacy highlights the need of personalized levothyroxine dosing to optimize therapeutic outcomes and minimize the risks of under- or overdosing in patients with low-risk DTC.

## Introduction

Differentiated thyroid cancer (DTC) is the most common endocrine malignancy. Its incidence has increased in recent years across all countries, reaching rates as high as 13 to 15 per 100,000 inhabitants per year (1, 2). The main causes of DTC, which includes papillary and follicular carcinoma, are genetic alterations that activate the MAPK pathway, especially mutations in BRAF (V600E) and RAS, as well as gene fusions such as RET and NTRK. Exposure to ionizing radiation in childhood is a well-established risk factor, and there are familial forms associated with genetic syndromes such as Cowden syndrome and familial adenomatous polyposis (3). Obesity and certain environmental exposures have been identified as potential risk factors, although their causal relationship requires further study (4).

Most DTCs display indolent behavior, with low mortality and excellent prognosis. Progression to more aggressive forms (poorly differentiated or anaplastic carcinoma) occurs through the accumulation of additional mutations (e.g., TP53, TERT, PI3K/AKT) that promote cellular immortalization and loss of differentiation (3, 5). The risk of recurrence and mortality is associated with variables such as age, tumor size, invasiveness, lymph node or distant metastasis, and specific molecular features (6). Most of these DTCs are classified as low risk. This group includes intrathyroidal tumors ≤4 cm in size, without aggressive histology, extrathyroidal invasion, or local or distant metastases (7, 8). In these tumors, progression is minimal and the prognosis is excellent, with a five-year survival rate of 98% (1, 9). Many of these tumors do not require aggressive treatment or intensive follow-up (10, 11).

For decades, treatment of DTC has been based on three main pillars: surgery, radioiodine (RAI), and inhibition of thyrotropin (TSH) secretion (12). The rationale for the latter is that most DTCs express TSH receptors, and therefore, the use of supraphysiological doses of thyroid hormone inhibits pituitary TSH release and is thought to suppress the proliferation and function of residual thyroid cells, both normal and cancerous (8). However, sustained suppression of TSH secretion is not harmless. Subclinical thyrotoxicosis resulting from excessive levothyroxine has been associated with an increased risk of atrial fibrillation, cardiovascular disease, osteoporosis, and fractures (12, 13).

The 2015 American Thyroid Association (ATA) clinical guideline (14) gives precise recommendations for TSH suppression in low-, intermediate-, and high-risk patients during the first year following initial treatment. One year after initial therapy, and at subsequent follow-up visits, patients should undergo re-stratification using dynamic risk stratification (DRS) and be classified according to their response to therapy (14–16). TSH should be maintained within the range of 0.5-2, between 0.1-0.5 and < 0.1 mcU/ml in the case of low risk patients who achieve an excellent, indeterminate or biochemical incomplete and structural incomplete response, respectively. Real-life studies show that the frequency of TSH suppression in patients with low-risk DTC is much higher than expected (17). A recent survey (18) of 448 North American endocrinologists and surgeons revealed that 48.8% of respondents strongly favored TSH suppression in patients with low-risk papillary thyroid cancer. However, multicenter studies specifically targeting patients at low risk of recurrence, who are the most common in clinical practice, are currently lacking. To give insight into the real-world approach of Spanish endocrinologists on this topic, we designed this study to assess the adequacy of levothyroxine therapy, evaluated by serum TSH levels, in patients with low-risk DTC both 12 months after initial treatment and at their final follow-up visit.

## Methods

### Subjects

This multicenter, retrospective study analyzed the adequacy of levothyroxine treatment in patients older than 18 years with a histologic diagnosis of low-risk DTC as defined by the ATA risk stratification system (14). To be included in the study, patients had to meet all of the following criteria: (a) surgical treatment by lobectomy or total/near total thyroidectomy, with or without lymph node dissection, followed or not by radioiodine (RAI) treatment, (b) availability of a histological report, (c) follow-up at the same hospital for at least 12 months, and (d) documentation of DRS at both 12 months and at the last clinical visit. Patients with incomplete follow-up data preventing the assessment of DRS were excluded.

### Study design

This project was disseminated through the Thyroid Task Force of the Spanish Society of Endocrinology and Nutrition (Sociedad Española de Endocrinología y Nutrición, SEEN) composed of endocrinologists with special expertise and dedicate ghion to thyroid disease. Seventeen investigators from fifteen hospital centers agreed to participate. Each investigator included adult patients with low-risk DTC who attended their outpatient clinics between January 1 and December 31, 2024.

Data collection encompassed clinical and demographic characteristics, initial surgery, RAI treatment when applicable, pathological findings, initial patient risk classification, and follow-up data, with particular attention on DRS assessment and serum TSH level obtained both at 12 months and at the last clinical visit. At the end of follow-up, additional data were collected on the presence of permanent postoperative hypoparathyroidism and any health conditions affecting levothyroxine dosage or TSH suppression. In particular, we consider the presence of osteoporosis, osteopenia, previous fracture, atrial fibrillation, tachycardia or other arrhythmias and fragility, since all these conditions can determine the degree of TSH suppression. For patients with a follow-up period exceeding two years, levothyroxine dose stability was assessed. Patients whose levothyroxine dose remained unchanged over the past year were classified stable, whereas those who had undergone one or more dose adjustments during that period were considered unstable. In patients considered unstable, various causal factors of variability in TSH determination were considered, such as lack of adherence to medication, pharmacological interferences, diseases that produce alterations in thyroxine absorption, significant changes in weight and other factors.

All patients’ data were obtained under the standard medical care conditions. This study was conducted in accordance with the Declaration of Helsinki (as revised in 2013). The patient’s confidential information was protected according to national law, and the study was approved by the local ethics committee of the Hospital Universitario Puerta de Hierro Majadahonda (Madrid, Spain) (PI 157/24).

### Initial classification of patients

For the classification of patients at the time of diagnosis, the tumor, node, metastases (TNM) staging system was used according to the criteria of the American Joint Committee on Cancer (AJCC/UICC), 8th edition (19). The initial risk of recurrence was classified according to the 2009 American Thyroid Association (ATA) criteria, with the modifications proposed in 2015 (14).

### Laboratory procedures

Serum thyroglobulin (Tg), Tg antibodies (TgAb) and TSH measurements were performed using standard procedures of each hospital. The instruments used for Tg and TSH quantification, along with the number of investigators and patients involved, are summarized in **Supplementary Tables S1** and **S2** (**Supplementary Material**). These tables also include the Tg detection limits for each of the assays used and the reference intervals for TSH provided by the manufacturers of the different analytical methods. TSH and other laboratory parameters were measured at participating centers in the morning, regardless of fasting status. The lower limit of the TSH reference interval ranged from 0.25 to 0.55 mcU/ml, while the upper limit was between 4.2 and 5.3 mcU/ml. All participating clinical laboratories used standardized methods and complied with established quality standards.

### Dynamic risk stratification

For the assessment of DRS, we considered biochemical data (Tg and TgAb) and imaging tests (ultrasound and others if necessary), as well as the treatment received by each patient (thyroidectomy plus RAI ablation, thyroidectomy without ablation and lobectomy) (14–16, 20, 21).

Tg determination was considered valid only when the TgAb titer was negative. Tg values were stratified into three groups as specified in **Supplementary Table S3** (**Supplementary Material**). In patients who underwent thyroidectomy plus RAI ablation, Tg levels were classified as follows: excellent response (Tg EX group) for values <0.20 ng/ml (<0.50 ng/ml for Immulite 2000); indeterminate response (Tg IN group) for values ​​between 0.20 and 0.99 ng/ml (0.5 to 0.99 ng/ml for Immulite 2000); and biochemical incomplete response (Tg BI group) for values ≥1 ng/ml. In patients who underwent thyroidectomy without ablation, the Tg EX, Tg IN and Tg BI groups were defined by Tg values ​​of <0.20, 0.20 to 4.99 and ≥5.0 ng/ml, respectively (<0.50, 0.50 to 4.99 and ≥5.0 ng/ml, respectively for Immulite 2000). For patients who underwent lobectomy, only two groups were defined: Tg EX (<30 ng/ml) and Tg BI (≥30 ng/ml). Thyroid ultrasound results were classified as negative (normal ultrasound), indeterminate (non-specific findings that cannot be classified as normal or suspicious) or suspicious (findings indicative of malignancy).

DRS was performed at 12 months and at the last follow-up visit. In both cases, patients were classified into four categories (15) based on the following criteria: excellent response (Tg EX, negative TgAb, and negative imaging), indeterminate response (Tg IN or non-rising positive TgAb, or indeterminate or suspicious imaging), biochemical incomplete response (Tg BI or rising TgAb), and structural incomplete response (evidence of structural or functional disease regardless of Tg or TgAb levels).

### Adequacy of levothyroxine treatment

The adequacy of levothyroxine treatment was assessed based on the TSH level at 12 months and at the last follow-up visit. In cases of structural incomplete response, a TSH level <0.1 mcU/ml was considered appropriate. In patients with an indeterminate or biochemical incomplete response, TSH values ​​between 0.1 and 0.5 mcU/ml were considered adequate. For patients with an excellent response, control was considered adequate when their serum TSH level was between 0.5 and 2.0 mcU/ml. When TSH levels were above the appropriate control range, it was considered that there was an insufficient dose of levothyroxine. TSH levels above the target range indicate insufficient levothyroxine dosing, whereas levels below the range indicated overtreatment.

### Statistical analysis

Quantitative variables are expressed as median (interquartile range, IQR). Categorical variables are described using absolute values, ratios, or percentages. Proportions were compared using the chi-square test or Fisher’s exact test. Comparisons of quantitative variables between two groups were conducting using the Mann–Whitney *U*-test. The McNemar test was used to compare treatment adequacy at 12 months and the last follow-up visit (paired data). Several univariate and multivariable logistic regression models were applied to identify factors influencing levothyroxine therapy adequacy at 12 months and at the last follow-up visit. We used variables that are generally associated with the prognosis of patients with DTC (gender, age, time of follow-up, histology, tumor size and incidental tumor), as well as variables that were significant in the comparative statistical analysis between patients with adequate and inadequate control (type of thyroidectomy, radioiodine, hypoparathyroidism, health problems and dose instability). All statistical tests were two-sided, and differences were considered significant when P <0.05.

## Results

### Patients’ characteristics

Of the 1168 patients with low-risk DTC selected for the study, 152 were excluded due to the lack of sufficient clinical information for DRS. The final cohort comprised 1016 patients (820 women, 80.7%) with median (IQR) age of 48 (38–59) years. Papillary thyroid carcinoma was diagnosed in 929 patients (91.4%), while 87 (8.6%) had follicular thyroid cancer. Surgical treatment included lobectomy in 81 patients (8.0%), total thyroidectomy in 610 cases (60.0%), and total thyroidectomy plus lymph node dissection in 325 (32.0%). RAI remnant ablation was administered to 667 patients (65.6%) postoperatively after initial surgery. Based on TNM staging, 94.7% (962) were classified as T1-T2 and 90.7% (922) as N0-Nx (**Table 1**).

**Table 1:** Clinical, pathological and therapeutic characteristics of the studied patients with low-risk differentiated thyroid carcinoma.

	Units or categories	No. (percentage)	Median (IQR)
Clinical			
Gender	Male	196 (19.3)	
	Female	820 (80.7)	
Age	Years		48 (38-59)
Family history		57 (5.6)	
HNRT in childhood		4 (0.4)	
Pathological			
Histology	Papillary	929 (91.4)	
	Follicular	87 (8.6)	
Tumor size	cm		1.2 (0.7-2.0)
Multifocality		372 (36.6)	
Chronic lymphocytic thyroiditis		291 (28.6)	
Incidental		242 (23.8)	
Therapeutic			
Surgery	Lobectomy	81 (8.0)	
	TT	610 (60.0)	
	TT + LND	325 (32.0)	
Radioiodine		667 (65.6)	
Risk classification			
T	T0-Tx	4 (0.4)	
	T1a	420 (41.3)	
	T1b	345 (34.0)	
	T2	197 (19.4)	
	T3	50 (4.9)	
N	N0-Nx	922 (90.7)	
	N1a	83 (8.2)	
	N1b	11 (1.1)	
TNM stage	I	984 (96.9)	
	II	31 (3.1)	
ATA risk	low	1016 (100)	

Data are the median (IQR) for quantitative variables, and the number (percentage) for categorical variables.

HNRT, head and neck radiation therapy in childhood; TT, total thyroidectomy; LND, lymph node dissection, ATA, American Thyroid Association; T, tumor; N, node.

### Follow-up and dynamic risk stratification

The median follow-up time was 79 (42–142) months. Health conditions potentially affecting levothyroxine dosing were identify in 88 patients (8.7%), including osteoporosis (51), atrial fibrillation (14), frailty (12), arrhythmia (7), and fractures (3). Among 898 patients with at least two years of follow-up, 310 (34.5%) exhibits dose instability. Identifiable causes were present in 194 cases, including weight changes (74), non-adherence (41), risk category change (24), poor tolerance or comorbidities (19), drug interactions (14), malabsorption (13), medication error (2) and other (7). No cause was identified in 116 patients. At the last visit evaluation, there were 83 patients (8.2%) with chronic hypoparathyroidism.

DRS outcomes at 12 months after initial treatment were as follows: excellent response, 62.3%; indeterminate response, 34.0%; incomplete biochemical response, 3.0%; and incomplete structural response, 0.8%. Final DRS outcomes were: excellent response, 77.8%; indeterminate response, 19.7%; incomplete biochemical response, 2.0%; and incomplete structural, 0.4% (**Table 2**).

**Table 2:** Dynamic risk stratification at 12 months after initial treatment and at the last visit.

	12 months (n=1016)	Last visit (n=978)
Group of thyroglobulin levels*		
Excellent (Tg EX)	700 (81.1 [78.4-83.6])	794 (87.4 [85.1-89.4])
Indeterminate (Tg IN)	133 (15.4 [13.2-18.0])	94 (10.4 [8.5-12.5])
Biochemical incomplete (TG BI)	30 (3.5 [2.5-4.9])	20 (2.2 [1.4-3.4])
Thyroglobulin antibodies		
Negative	863 (84.9 [82.6-87.0])	908 (92.8 [91.1-94.3])
Positive	153 (15.1 [13.0-17.4])	70 (7.2 [5.7-9.0])
Ultrasound		
Negative	873 (85.9 [83.7-87.9])	584 (59.7 [56.6-62.7])
Doubtful	122 (12.0 [10.2-14.2])	71 (7.3 [5.8-9.1])
Suspicious	8 (0.8 [0.4-1.6])	4 (0.4 [0.2-1.1])
Not performed	13 (1.3 [0.8-2.2])	319 (32.6 [29.8-35.6])
Dynamic risk stratification		
Excellent	633 (62.3 [59.3-65.2])	761 (77.8 [75.1-80.3])
Indeterminate	345 (34.0 [31.1-36.9])	193 (19.7 [17.4-22.3])
Biochemical incomplete	30 (3.0 [2.1-4.2])	20 (2.0 [1.3-3.1])
Structural incomplete	8 (0.8 [0.4-1.6])	4 (0.4 [0.2-1.1])

Data are the number (percentage and 95% confidence interval).

*Valid thyroglobulin measurements (negative thyroglobulin antibodies) were 863 at 12 months and 908 at last visit.

### Adequacy of levothyroxine treatment

Twelve months after initial therapy, 264 patients (26.0%) had TSH levels within the target for their DRS status, indicating adequate control. At the final evaluation, this proportion increased significantly to 39.6% (387/978; P <0.001; McNemar test). The adequacy of treatment across DRS categories is summarized in **Table 3**.

**Table 3:** Number and proportion of patients with adequate and inadequate control at 12 months and at the last visit.

	All	Excellent	Indeterminate	Biochemical incomplete	Structural incomplete
Adequacy at 12 months					
Number of patients	1016	633	345	30	8
Adequate	264 (26.0 [23.4-28.8])	163 (25.8 [22.5-29.3])	94 (27.2 [22.8-32.2])	4 (13.3)	3 (37.5)
Excess	485 (47.7 [44.7-50.8])	391 (61.8 [57.9-65.5])	84 (24.3 [20.1-29.1])	10 (33.3 [19.2-51.2])	0
Insufficient	267 (26.3 [23.7-29.1])	79 (12.5 [10.1-15.3])	167 (48.4 [43.2-53.7])	16 (53.3 [36.1-70.0])	5 (62.5)
Adequacy at last visit					
Number of patients	978	761	193	20	4
Adequate	387 (39.5 [33.6-42.7])	337 (44.3 [40.8-47.8])	46 (23.8 [18.4-30.3])	3 (15.0)	1 (25.0)
Excess	266 (27.2 [24.5-30.1])	232 (30.5 [27.3-33.9])	27 (14.0 [9.8-19.6])	7 (35.0 [0.18-0.57])	0
Insufficient	325 (33.2 [30.4-36.2])	192 (25.2 [22.3-28.4])	120 (62.2 [55.2-68.7])	10 (50.0 [0.30-0.71])	3 (75.0)

Data are the number (percentage and 95% confidence interval).

**Figure 1:**
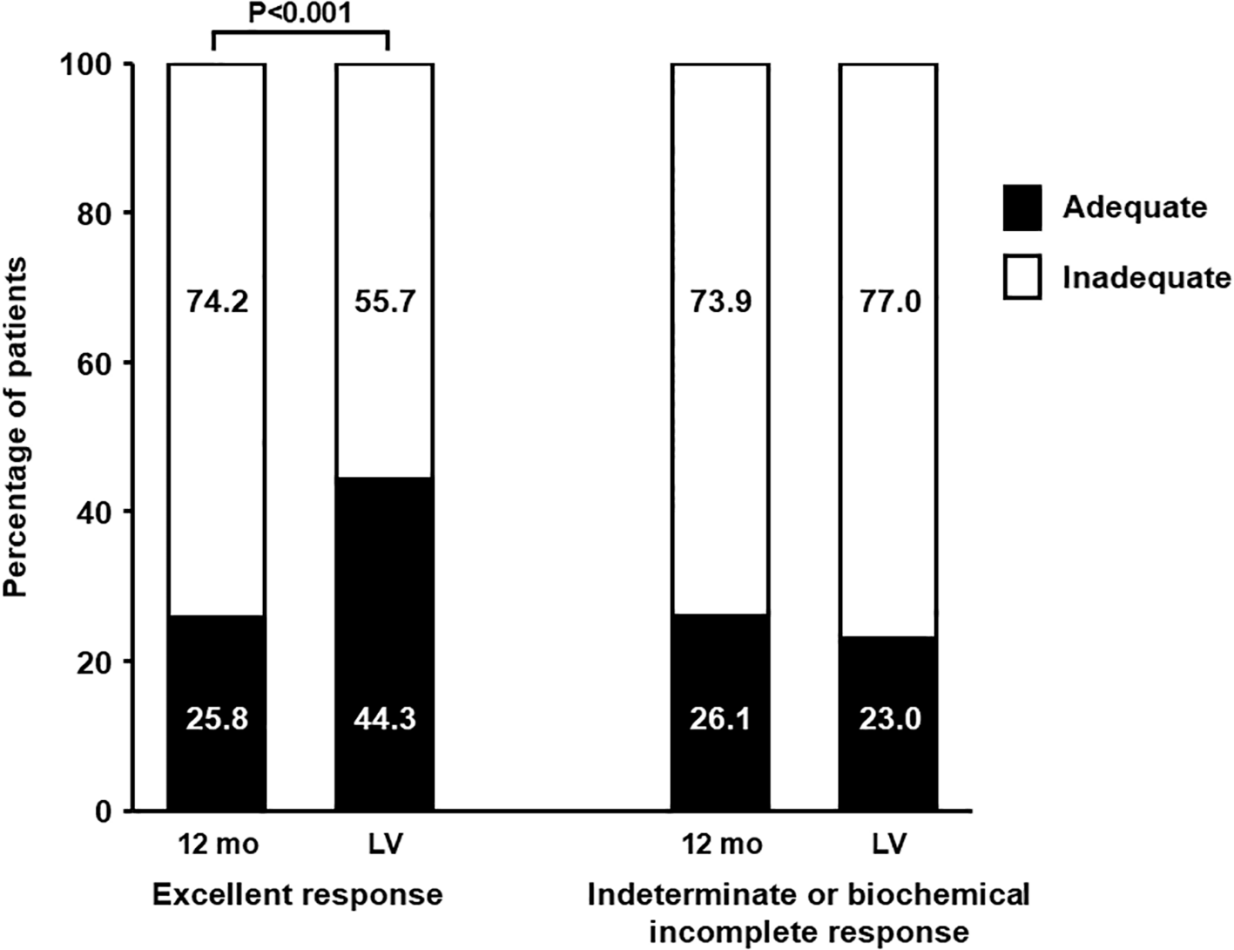
Percentage of patients with adequate and inadequate control at 12 months after initial treatment and at the final visit. Patients are classified according to response type (excellent, and indeterminate or biochemical incomplete response).

For comparative analysis, two groups of patients were examined: those with excellent response versus those with indeterminate or incomplete biochemical response. Patients with structural incomplete response were excluded due to their distinct characteristics and small number. Among patients with excellent response, treatment adequacy increased significantly from 25.8% at 12 months to 44.3% at the last visit (P<0.001; **Figure 1**). However, no significant changes in adequacy were observed over time in the indeterminate or incomplete biochemical response group (26.1% vs 23.0%; P = 0.515).

**Table 4** shows characteristics of patients with excellent response and adequate control compared to patients with inadequate control at 12 months. Patients with adequate control were more likely to be male (25.8 vs. 17.4%; P = 0.029), have incidental tumors (27.8 vs. 19.4%; P = 0.027), and have hypoparathyroidism (11.7 vs. 6.0%; P = 0.023). Conversely, they had lower rates of total thyroidectomy (88.3 vs. 93.8%; P = 0.026) and RAI treatment (55.2 vs. 75.1%; P<0.001). At the last visit, treatment adequacy was significantly associated with dose instability, which was higher in inadequately controlled patients (49.2 vs. 12.0%, P<0.001).

**Table 4:** Comparison of patients with adequate and inadequate control at 12 months and at the last follow-up visit (patients with excellent response).

	Adequacy of levothyroxine therapy in patients with excellent response					
	Evaluation at 12 months			Evaluation at last visit		
	Adequate (n=167)	Inadequate (n=470)	P value	Adequate (n=337)	Inadequate (n=424)	P value
Gender			0.029			0.136
Male	42 (25.8)	82 (17.4)		72 (21.4)	72 (17.0)	
Female	121 (74.2)	388 (82.6)		265 (78.6)	352 (83.0)	
Age, yr	48 (39-58)	48 (38-59)	0.764	47 (37-58)	48 (38-58)	0.613
Time of follow-up, mo	—	—		101 (58-160)	84 (49-143)	0.011
Histology			0.638			0.214
Papillary	147 (90.2)	429 (91.3)		300 (89.0)	389 (91.7)	
Follicular	16 (9.8)	41 (8.7)		37 (11.0)	35 (8.3)	
Tumor size, cm	1.2 (0.6-2.0)	1.3 (0.8-2.2)	0.148	1.3 (0.7-2.3)	1.3 (0.8-2.0)	0.393
Multifocal	55 (33.7)	177 (37.3)	0.397	119 (35.3)	167 (39.4)	0.259
CLT	44 (27.0)	118 (25.1)	0.677	85 (25.3)	127 (30.0)	0.167
Incidental	45 (27.8)	91 (19.4)	0.027	79 (23.5)	88 (20.8)	0.379
Surgery			0.026			0.454
Lobectomy	19 (11.7)	29 (6.2)		24 (7.1)	24 (5.7)	
Total thyroidectomy	144 (88.3)	441 (93.8)		313 (92.9)	400 (94.3)	
Radioiodine	90 (55.2)	353 (75.1)	<0.001	247 (73.3)	309 (72.9)	0.935
TNM			1.0			0.167
I	157 (96.3)	453 (96.4)		322 (95.5)	413 (97.4)	
II	6 (3.7)	17 (3.6)		15 (4.5)	11 (2.6)	
Hypoparathyroidism	19 (11.7)	28 (6.0)	0.023	25 (7.4)	34 (8.0)	0.787
Health problem	—	—		29 (9.0)	41 (9.7)	0.705
Dose instability*	—	—		40 (12.0)	203 (49.2)	<0.001

Data are the median (IQR) for quantitative variables, and the number (percentage) for categorical variables.

CLT, chronic lymphocytic thyroiditis; TNM, tumor-node-metastasis staging system.

*At the last visit, the number of patients with more than 2 years of follow-up was 706 (322 with adequate control and 384 with inadequate control).

Patients with indeterminate and biochemical incomplete response were analyzed together (**Table 5**). At 12 months patients with adequate control had higher rates of follicular carcinoma (13.3 vs. 5.8%; P = 0.026), total thyroidectomy (99.0 vs. 88.4%; P = 0.001) and RAI treatment (72.4 vs. 53.1%; P = 0.001) compared with patients with inadequate control. They also had larger tumors (1.5 [0.8-2.0] vs. 1.0[0.6-1.8] cm; P = 0.021). At the end of follow-up, patients with adequate control had larger tumors than those with inadequate control (1.3 [0.8-2.2] vs. 0.9 [0.5-1.5] cm; P = 0.017), while no differences were observed in other variables.

**Table 5:** Comparison of patients with adequate and inadequate control at 12 months and at the last follow-up visit (patients with indeterminate or biochemical incomplete response).

	Adequacy of levothyroxine therapy in patients with indeterminate or biochemical incomplete response					
	Evaluation at 12 months			Evaluation at last visit		
	Adequate (n=98)	Inadequate (n=277)	P value	Adequate (n=49)	Inadequate (n=164)	P value
Gender			0.367			1.0
Male	15 (15.3)	55 (19.9)		10 (20.4)	33 (20.1)	
Female	83 (84.7)	222 (80.1)		39 (79.6)	131 (79.9)	
Age, yr	48 (40-60)	48 (39-58)	0.629	52 (40-63)	51 (43-62)	0.570
Time of follow-up, mo	—	—		69 (37-92)	58 (35-92)	0.809
Histology			0.026			0.700
Papillary	85 (86.7)	261 (94.2)		46 (93.9)	157 (95.7)	
Follicular	13 (13.3)	16 (5.3)		3 (6.1)	7 (4.3)	
Tumor size, cm	1.5 (0.8-2.0)	1.0 (0.6-1.8)	0.021	1.3 (0.8-2.2)	0.9 (0.5-1.5)	0.017
Multifocal	37 (37.8)	101 (36.5)	0.903	14 (28.6)	60 (36.6)	0.393
CLT	32 (32.7)	95 (34.5)	0.804	17 (34.7)	53 (32.5)	0.863
Incidental	22 (22.4)	83 (30.2)	0.153	12 (24.5)	58 (35.6)	0.268
Surgery			0.001			0.457
Lobectomy	1 (1.0)	31 (11.6)		4 (8.2)	22 (13.4)	
Total thyroidectomy	97 (99.0)	245 (88.4)		45 (91.8)	142 (86.6)	
Radioiodine	71 (72.4)	147 (53.1)	0.001	24 (49.0)	66 (40.2)	0.324
TNM			0.056			0.545
I	93 (94.9)	273 (98.6)		48 (98.0)	162 (98.8)	
II	5 (5.1)	4 (1.4)		1 (2.0)	2 (1.2)	
Hypoparathyroidism	7 (7.1)	27 (9.7)	0.542	4 (8.2)	16 (9.8)	1.0
Health problem				5 (10.2)	12 (7.8)	0.564
Dose instability*	—	—		15 (32.6)	74 (46.8)	0.094

Data are the median (IQR) for quantitative variables, and the number (percentage) for categorical variables.

CLT, chronic lymphocytic thyroiditis; TNM, tumor-node-metastasis staging system.

*At the last visit, the number of patients with more than 2 years of follow-up was 188 (45 with adequate control and 143 with inadequate control).

### Multivariable logistic regression analysis

**Figure 2:**
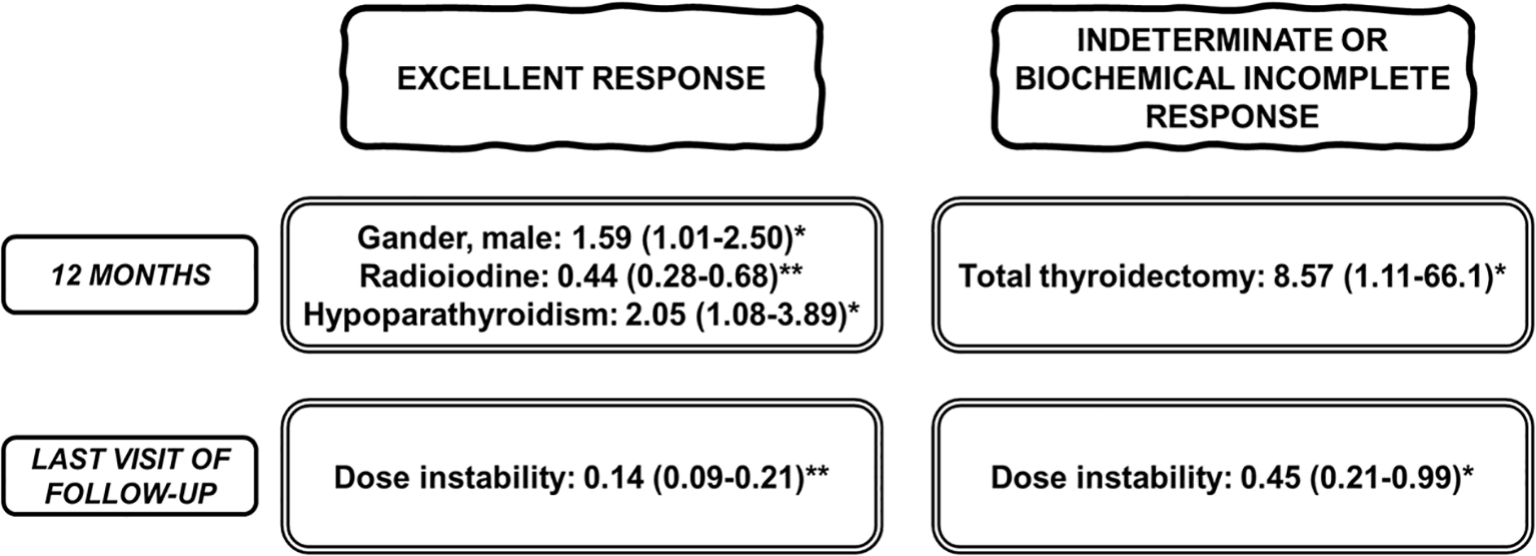
Summary of the results of the multivariate logistic regression analysis (model 2) to study the influence of several variables on the adequacy of levothyroxine treatment. Patients are classified according to treatment response (excellent response and indeterminate or biochemical incomplete response) both at 12 months and at the last follow-up visit. Data are the odds ratio with 95% confidence intervals. *P < 0.05, **P < 0.001.

Results of several models of multivariable logistic regression analysis are shown in **Supplementary Material** (**Supplementary Tables S4**-**S7**). A summary of the most relevant findings is shown in **Figure 2**. At 12 months treatment adequacy was significantly associated with male sex and hypoparathyroidism in patients with excellent response and with total thyroidectomy in those with indeterminate or incomplete biochemical response. RAI treatment showed significant but inverse association with adequacy in the excellent response group. In the last visit adequacy was inversely correlated with dose instability in both excellent, indeterminate and biochemical incomplete response.

## Discussion

This study examines the adequacy of levothyroxine treatment in a large cohort of patients with low-risk DTC followed by expert endocrinologists. Three key findings emerge: first, the proportion of patients achieving adequate control is strikingly low; second, treatment adequacy improves at the last follow-up, but only in patients with excellent response; and third, levothyroxine dose instability is the main determinant of long-term inadequacy.

### Low adequacy of levothyroxine treatment

Our results show a low level of levothyroxine therapy adequacy, with only 26.0% of patients within the target TSH range at 12 months of initial treatment and 39.5% at the end of the follow-up period. In an effort to decrease unnecessary adverse effects of overdose of levothyroxine, national (22) and international (14) clinical guidelines clearly propose a graded tailored approach to TSH suppression based on initial risk of recurrence, and subsequent response to therapy.

Accordingly, the results obtained in thyroid cancer patients in Spain seem discouraging. A previous study in 216 patients with DTC (69.2% of them low-risk) found that 30.7% of low-risk patients with an excellent response had unnecessarily suppressed TSH levels, though this percentage decreased to 16.3% after a mean follow-up of 6.9 years (23). Another study of 102 low-risk DTC patients reported that only 8.8% had appropriate TSH levels at 12 months, improving to 19.6% after a mean follow-up of 5.9 years (24). A multicenter study in Turkey (25), including 1125 patients with all-risk DTC, found that only 29.2% of patients achieved adequate target TSH levels with overtreatment observed in 50.4% of all analyzed patients and 80.2% of low-risk cases, whereas undertreatment was detected in 20.4%. Conversely, a Chinese study reported a 61.4% adequacy rate (26), though it included only intermediate- and high-risk DTC patients and had a follow-up of just one year.

In short, our findings reinforce the conclusion that levothyroxine overuse is prevalent particularly in patients with excellent response. However, overtreatment is not unique to thyroid cancer; it has also been reported in patients with subclinical hypothyroidism with the intention of alleviating symptoms or improving quality of life, despite a lack of supporting evidence (27).

### Improvement in treatment adequacy over time

The proportion of well-controlled patients increased by the end of the follow-up compared to the 12-month visit; however, this improvement was observed only in patients with excellent response and not in other types of responses. In our study 62.3% of low-risk patients had an excellent response at 12-month increasing to 77.8% at the final visit. These results are comparable, though slightly lower, than those reported by other authors where excellent response rates range from 70 to 91% (14, 28, 29). The increase in the excellent response rate between the 12-month and the final assessments also suggests a corresponding decrease in the proportion of patients with less-than-excellent responses decreases over time, indicating that treatment intensity should be reduced in many of them. These results are consistent with those of other authors who have reported a favorable evolution of patients with an excellent response (29). In relation to these considerations, the improvement in the adequacy of levothyroxine treatment observed in this study could be related to the increase in the proportion of patients with an excellent response between the 12-month evaluation and the last visit, since the TSH target range for these patients is broader than that for patients with less than excellent responses. Other factors that could explain the observed improvement in TSH adequacy over time include better patient adherence to the prescribed treatment, greater diligence by physicians in adjusting levothyroxine doses, or increased frequency of laboratory monitoring.

The 2015 ATA guidelines recommend maintaining TSH levels between 0.5 to 2.0 mcU/ml for patients with an excellent response. However, only 25.8% of our patients achieved this target at 12 months, increasing to 44.3% at the final follow-up visit. Among patients with excellent response, inadequate control of TSH levels was primarily due to levothyroxine overdosing, whereas, in those with indeterminate response, it was more often due to insufficient dosing (**Table 3**). These data suggest a tendency among Spanish endocrinologists to favor excessive TSH suppression in patients with an excellent response, while being reluctant to lower TSH below 0.5 mcU/ml in patients with indeterminate or incomplete biochemical responses.

Therapeutic inertia in reducing levothyroxine dosage in patients with excellent response may stem from concerns about cancer recurrence if TSH suppression is relaxed (18, 30). Nonetheless, there is no evidence that aggressive TSH suppression reduces recurrence in low-risk patients (17, 31). Moreover, long-term TSH suppression is associated with serious health risks, including cardiac morbidity, bone loss, fractures, and impaired quality of life (13, 32). Furthermore, Qiang et al. (33) recently found that maintaining TSH in the range of 0.5–2 mIU/L showed no significant difference in DTC recurrence compared with TSH levels of 2–4 mIU/L, suggesting that guidelines may consider liberalizing TSH targets in low-risk cohorts.

### Dose instability as the key determinant of inadequate treatment

Our multivariate analysis identified gender, RAI, and hypoparathyroidism as predictors of treatment adequacy at 12-months in patients with an excellent response, while total thyroidectomy was a determinant for patients with a less favorable response. These associations are challenging to interpret; however, they highlight the fact that classic histological variables (multifocality, vascular invasion, tumor size) were not significant in the multivariate analysis. These findings are consistent with previous reports by other authors (23). Nevertheless, caution should be exercised when interpreting these associations, as the design of our study does not allow us to establish causal relationships.

At the last visit, dose instability emerged as the most important and significant factor determining treatment adequacy. A strong inverse relationship was observed between instability and treatment adequacy, especially in patients with excellent response (HR 0.14), but also in those with suboptimal responses (HR 0.45). Dose instability was observed in 310 of 898 patients (34.5%) with over two years of follow-up. Identifiable causes included weight fluctuations, medication non-adherence, changes in risk classification, drug interaction, and malabsorption (34).

Poor adherence was detected in 13% (41 of 313) of our patients. This is a common cause of treatment inadequacy in various chronic conditions, including hypothyroidism (35, 36). Our results are similar to those reported by Yavuz et al. (25), who found that 16.4% of patients missed at least one dose in the past month. Other studies have reported even higher non-adherence rates, with 52% of thyroid cancer classified as non-adherent in one survey (37).

Additionally, 37.4% of our patients require dose adjustments for unidentified reasons, possibly due to spontaneous TSH variations. Previous studies indicate that up to 20% athyreotic patients exhibit significant spontaneous TSH variations despite stable levothyroxine dosing (28). Given the narrow therapeutic index of levothyroxine, small dose adjustments can result in significant TSH fluctuations, impacting serum Tg levels and DRS classification (38, 39), especially in low-risk DTC treated with thyroidectomy without RAI (40).

### Clinical implications

Our study may have important clinical implications as we are currently experiencing an era of overdiagnosis of DTC due to the widespread availability and use of ultrasound and other imaging tests. This has led to an unprecedented rise in the incidence of these tumors (41). Overdiagnosis is often followed by overtreatment, which carries potential health risks and contributes to the excessive consumption of healthcare resources (42).

Our findings suggest that Spanish endocrinologists struggle to adhere to clinical guidelines. Various reasons have been put forward for clinicians’ failure to follow guidelines, including lack of familiarity, disagreement with recommendations, inertia of previous practice, or difficulty in reconciling patient preferences with guideline recommendations (43). Furthermore, some clinicians may fail to reassess their patients’ recurrence risk or may not fully recognize the appropriate TSH targets according to guideline-based risk stratification, further contributing to suboptimal management. In the case of low-risk DTC, clinicians should try to avoid levothyroxine overdosage in patients with excellent response. It has been well established that iatrogenic thyrotoxicosis is associated with the various above-mentioned health risks and also extra laboratory monitoring and physician visits (12, 13). Therefore, we believe that proper levothyroxine dose adjustment is essential at all visits in patients with thyroid cancer, and that the TSH targets established by guidelines should be pursued. In addition, patients with dose instability deserve special attention, and efforts should be made not only to determine the cause of the instability but also to take measures to prevent TSH fluctuations and continuous dose changes in these patients.

One final consideration should be added to our comments. It is true that while TSH suppression therapy has traditionally been regarded as an adjunctive treatment following thyroid cancer surgery, there remains an ongoing clinical question as to whether such therapy is genuinely necessary for patients with low-risk DTC. This suppression was based on the fact that DTC tumor cells express TSH receptors and respond to their stimulation with cell proliferation. Therefore, supraphysiological doses of levothyroxine have historically been used as adjuvant therapy after surgery, with the aim of reducing the risk of recurrence or tumor progression (12). However, current evidence has substantially modified this practice, especially in patients with low-risk DTC. The latest ATA guidelines (44), published after this study was conducted, do not recommend prolonged TSH suppression in patients with low- or intermediate-risk disease who do not have evidence of biochemical or structural recurrence. The rationale is that these patients have an excellent prognosis, and TSH suppression does not provide additional benefits in terms of recurrence or overall survival.

### Strengths and limitations

The main strengths of our study include its large sample size, multicenter national setting, well-defined inclusion criteria, and rigorous data collection by specialists in thyroid disease. All data were obtained from standard practice at the participating centers, ensuring that the findings reflect real-life outcomes in thyroid cancer patients treated in Spain.

However, this study has limitations inherent to its retrospective nature. Follow-up protocols may have varied across centers and differences in laboratory measurements methods could affect comparability of serum Tg data. Therefore, we consider that future research would benefit from prospective cohort designs with pre-specified data collection protocols, ensuring standardized and complete documentation of clinical, biochemical, and treatment-related variables. Pragmatic randomized designs evaluating different TSH targets or levothyroxine management strategies could further reduce confounding. Furthermore, future multicenter studies should adopt harmonized follow-up protocols or, at minimum, collect detailed data on center-specific practices to adjust for these differences analytically. Whenever possible, future studies should standardize laboratory methods by using uniform assays or perform cross-calibration studies.

On the other hand, our study has the inherent limitation of excluding a group of patients with insufficient clinical information, which may have led to potential selection bias and a lack of longitudinal trend analysis of TSH. Prospective designs with standardized data collection and employing longitudinal TSH data will minimize missing information. Expanding the data sources (e.g., linking electronic health records with pharmacy and administrative databases) may also reduce the proportion of patients with incomplete information. Together, these limitations highlight the challenges of assessing adequacy of levothyroxine therapy in retrospective, multicenter settings. Prospective study designs, standardized protocols, and robust longitudinal analyses would substantially enhance the reliability and generalizability of future findings.

## Conclusions

In conclusion, the adequacy of levothyroxine treatment in patients with low-risk DTC remains suboptimal in the studied cohort. Our findings indicate that, after a mean follow-up of 6.6 years, 30.5% of patients with excellent response are overtreated with supraphysiological doses of levothyroxine, while 62% of those with an indeterminate response and 50% of those with an incomplete biochemical response receive insufficient doses of thyroid hormone. Consequently, the former faces an increased risk of complications associated with subclinical thyrotoxicosis, whereas the latter may have a heightened risk of tumor recurrence. Preventing both overtreatment and undertreatment is mandatory, requiring greater adherence to clinical guidelines by the clinicians managing these patients.

The potential lessons for clinical practice derived from the findings of our study can be summarized in the following points: (a) clinicians should overcome barriers to adherence to clinical guidelines, (b) levothyroxine doses should be adjusted at all visits of patients with DTC, and (c) special attention should be paid to patients with dose instability. It is our opinion that these recommendations would serve to improve the adequacy of healthcare for patients with DTC. Achieving optimal therapeutic adequacy requires a personalized approach to treatment, considering each patient’s risk of recurrence and response to treatment, as assessed by the DRS at each follow-up visit.

## Data Availability

The raw data supporting the conclusions of this article will be made available by the authors, without undue reservation.

## References

[1] Davies L Welch HG . Current thyroid cancer trends in the United States. JAMA Otolaryngol Head Neck Surg. (2014) 140:317–22. doi: 10.1001/jamaoto.2014.1, PMID: 24557566

[2] Powers AE Marcadis AR Lee M Morris LGT Marti JL . Changes in trends in thyroid cancer incidence in the United States, 1992 to 2016. JAMA. (2019) 322:2440–1. doi: 10.1001/jama.2019.18528, PMID: 31860035 PMC6990659

[3] Fagin JA Krishnamoorthy GP Landa I . Pathogenesis of cancers derived from thyroid follicular cells. Nat Rev Cancer. (2023) 23:631–50. doi: 10.1038/s41568-023-00598-y, PMID: 37438605 PMC10763075

[4] Peng G Pan X Ye Z Yi X Xie Q Zhang X . Nongenetic risk factors for thyroid cancer: an umbrella review of evidence. Endocrine. (2025) 88:60–74. doi: 10.1007/s12020-024-04155-x, PMID: 39745600

[5] Leandro-García LJ Landa I . Mechanistic insights of thyroid cancer progression. Endocrinology. (2023) 164:bqad118. doi: 10.1210/endocr/bqad118, PMID: 37503738 PMC10403681

[6] Fagin JA Wells SA Jr. Biologic and clinical perspectives on thyroid cancer. N Engl J Med. (2016) 375:1054–67. doi: 10.1056/NEJMra1501993, PMID: 27626519 PMC5512163

[7] Lamartina L Leboulleux S Terroir M Hartl D Schlumberger M . An update on the management of low-risk differentiated thyroid cancer. Endocr Relat Cancer. (2019) 26:R597–610. doi: 10.1530/ERC-19-0294, PMID: 31484161

[8] Schlumberger M Leboulleux S . Current practice in patients with differentiated thyroid cancer. Nat Rev Endocrinol. (2021) 17:176–88. doi: 10.1038/s41574-020-00448-z, PMID: 33339988

[9] Tran TV Schonfeld SJ Pasqual E Haymart MR Morton LM Kitahara CM . All-cause and cause-specific mortality among low-risk differentiated thyroid cancer survivors in the United States. Thyroid. (2024) 34:215–24. doi: 10.1089/thy.2023.0449, PMID: 38149602 PMC10884550

[10] Park JH Yoon JH . Lobectomy in patients with differentiated thyroid cancer: indications and follow-up. Endocr Relat Cancer. (2019) 26:R381–93. doi: 10.1530/ERC-19-0085, PMID: 31018176

[11] Ito Y Miyauchi A Fujishima M . Active surveillance for small papillary thyroid carcinoma. Endocr Relat Cancer. (2025) 32:e250287. doi: 10.1530/ERC-25-0287, PMID: 41196684

[12] Biondi B Cooper DS . Thyroid hormone suppression therapy. Endocrinol Metab Clin North Am. (2019) 48:227–37. doi: 10.1016/j.ecl.2018.10.008, PMID: 30717904

[13] Lee SY Pearce EN . Hyperthyroidism: A review. JAMA. (2023) 330:1472–83. doi: 10.1001/jama.2023.19052, PMID: 37847271 PMC10873132

[14] Haugen BR Alexander EK Bible KC Doherty GM Mandel SJ Nikiforov YE . 2015 American thyroid association management guidelines for adult patients with thyroid nodules and differentiated thyroid cancer: the American thyroid association guidelines task force on thyroid nodules and differentiated thyroid cancer. Thyroid. (2016) 26:1–133. doi: 10.1089/thy.2015.0020, PMID: 26462967 PMC4739132

[15] Tuttle RM Tala H Shah J Leboeuf R Ghossein R Gonen M . Estimating risk of recurrence in differentiated thyroid cancer after total thyroidectomy and radioactive iodine remnant ablation: using response to therapy variables to modify the initial risk estimates predicted by the new American Thyroid Association staging system. Thyroid. (2010) 20:1341–9. doi: 10.1089/thy.2010.0178, PMID: 21034228 PMC4845674

[16] Vaisman F Tala H Grewal R Tuttle RM . In differentiated thyroid cancer, an incomplete structural response to therapy is associated with significantly worse clinical outcomes than only an incomplete thyroglobulin response. Thyroid. (2011) 21:1317–22. doi: 10.1089/thy.2011.0232, PMID: 22136267

[17] Gigliotti BJ Jasim S . Differentiated thyroid cancer: a focus on post-operative thyroid hormone replacement and thyrotropin suppression therapy. Endocrine. (2024) 83:251–8. doi: 10.1007/s12020-023-03548-8, PMID: 37824045

[18] Papaleontiou M Chen DW Banerjee M Reyes-Gastelum D Hamilton AS Ward KC . Thyrotropin suppression for papillary thyroid cancer: A physician survey study. Thyroid. (2021) 31:1383–90. doi: 10.1089/thy.2021.0033, PMID: 33779292 PMC8558057

[19] Tuttle MM Haugen B Shah J Sosa JA Rohren E Subramaniam RM . Thyroid differentiated and anaplastic carcinoma. In: Amin MB Edge SB Greene F Byrd DR Brookland RK Washington MK , editors. AJCC Cancer Staging Manual. 8th ed. Springer International, New York (2017).

[20] Momesso DP Vaisman F Yang SP Bulzico DA Corbo R Vaisman M . Dynamic risk stratification in patients with differentiated thyroid cancer treated without radioactive iodine. J Clin Endocrinol Metab. (2016) 101:2692–700. doi: 10.1210/jc.2015-4290, PMID: 27023446 PMC6287503

[21] Vaisman F Momesso D Bulzico DA Pessoa CH Dias F Corbo R . Spontaneous remission in thyroid cancer patients after biochemical incomplete response to initial therapy. Clin Endocrinol (Oxf). (2012) 77:132–8. doi: 10.1111/j.1365-2265.2012.04342.x, PMID: 22248037

[22] Díez JJ Oleaga A Álvarez-Escolá C Martín T Galofré JC . Clinical guideline for management of patients with low risk differentiated thyroid carcinoma. Endocrinol Nutr. (2015) 62:e57–72. doi: 10.1016/j.endonu.2015.02.006, PMID: 25857691

[23] Díaz-Soto G Fernández-Velasco P Torres Torres B López Gómez JJ García Calvo S de Luis Román D . Evolution of suppressing TSH therapy at diagnosis and in the long-term follow-up in a cohort of differentiated thyroid cancer. Endocrinol Diabetes Nutr (Engl Ed). (2022) 69:844–51. doi: 10.1016/j.endien.2022.11.031, PMID: 36470820

[24] Thewjitcharoen Y Chatchomchuan W Wanothayaroj E Butadej S Nakasatien S Krittiyawong S . Clinical inertia in thyrotropin suppressive therapy for low-risk differentiated thyroid cancer: A real-world experience at an endocrine center in Bangkok. Med (Baltimore). (2024) 103:e38290. doi: 10.1097/MD.0000000000038290, PMID: 38788029 PMC11124651

[25] Yavuz DG Yazan CD Hekimsoy Z Aydin K Gokkaya N Ersoy C . Assesment of attainment of recommended TSH levels and levothyroxine compliance in differentiated thyroid cancer patients. Clin Endocrinol (Oxf). (2022) 97:833–40. doi: 10.1111/cen.14787, PMID: 35639050

[26] Ming J Zhu JQ Zhang H Sun H Wang J Cheng RC . A multicenter, prospective study to observe the initial management of patients with differentiated thyroid cancer in China (DTCC study). BMC Endocr Disord. (2021) 21:208. doi: 10.1186/s12902-021-00871-x, PMID: 34670546 PMC8529744

[27] Brito JP Ross JS El Kawkgi OM Maraka S Deng Y Shah ND . Levothyroxine use in the United States, 2008-2018. JAMA Intern Med. (2021) 181:1402–5. doi: 10.1001/jamainternmed.2021.2686, PMID: 34152370 PMC8218227

[28] Grani G Tumino D Ramundo V Ciotti L LoMonaco C Armillotta M . Changes in TSH levels in athyreotic patients with differentiated thyroid cancer during levothyroxine therapy: influence on dose adjustments. J Endocrinol Invest. (2019) 42:1485–90. doi: 10.1007/s40618-019-01074-x, PMID: 31203497

[29] Volpi F Alcalde J Larrache J Alegre E Argueta A Lozano MD . Tracking dynamic evolution of low- and intermediate-risk differentiated thyroid cancer: Identification of individuals at risk of recurrence. Clin Endocrinol (Oxf). (2024) 101:286–94. doi: 10.1111/cen.15111, PMID: 39038163

[30] Evron JM Reyes-Gastelum D Banerjee M Scherer LD Wallner LP Hamilton AS . Role of patient maximizing-minimizing preferences in thyroid cancer surveillance. J Clin Oncol. (2019) 37:3042–9. doi: 10.1200/JCO.19.01411, PMID: 31573822 PMC6839910

[31] Jonklaas J Sarlis NJ Litofsky D Ain KB Bigos ST Brierley JD . Outcomes of patients with differentiated thyroid carcinoma following initial therapy. Thyroid. (2006) 16:1229–42. doi: 10.1089/thy.2006.16.1229, PMID: 17199433

[32] Wang LY Smith AW Palmer FL Tuttle RM Mahrous A Nixon IJ . Thyrotropin suppression increases the risk of osteoporosis without decreasing recurrence in ATA low- and intermediate-risk patients with differentiated thyroid carcinoma. Thyroid. (2015) 25:300–7. doi: 10.1089/thy.2014.0287, PMID: 25386760 PMC6916125

[33] Qiang JK Sutradhar R Everett K Eskander A Lega IC Zahedi A . Association between serum thyrotropin and cancer recurrence in differentiated thyroid cancer: A population-based retrospective cohort study. Thyroid. (2025) 35:208–15. doi: 10.1089/thy.2024.0330, PMID: 39723994

[34] Jonklaas J Bianco AC Bauer AJ Burman KD Cappola AR Celi FS . Guidelines for the treatment of hypothyroidism: prepared by the American Thyroid Association Task Force on thyroid hormone replacement. Thyroid. (2014) 24:1670–751. doi: 10.1089/thy.2014.0028, PMID: 25266247 PMC4267409

[35] Vezzani S Giannetta E Altieri B Barbonetti A Bellastella G Certo R . An Italian survey of compliance with major guidelines for L-thyroxine of primary hypothyroidism. Endocr Pract. (2018) 24:419–28. doi: 10.4158/EP-2017-0159, PMID: 29847168

[36] Cappelli C Castello R Marini F Paoletta A Marchetti M Saullo M . Adherence to levothyroxine treatment among patients with hypothyroidism: A Northeastern Italian survey. Front Endocrinol (Lausanne). (2018) 9:699. doi: 10.3389/fendo.2018.00699, PMID: 30532737 PMC6265311

[37] Mols F Thong M Denollet J Oranje WA Netea-Maier RT Smit JWA . Are illness perceptions, beliefs about medicines and Type D personality associated with medication adherence among thyroid cancer survivors? A study from the population-based PROFILES registry. Psychol Health. (2020) 35:128–43. doi: 10.1080/08870446.2019.1619730, PMID: 31130004

[38] Flinterman LE Kuiper JG Korevaar JC van Dijk L Hek K Houben E . Impact of a forced dose-equivalent levothyroxine brand switch on plasma thyrotropin: A cohort study. Thyroid. (2020) 30:821–8. doi: 10.1089/thy.2019.0414, PMID: 32188356

[39] Razvi S Nicodemus N Ratnasingam J Arundhati D Soh WEA Kunavisarut T . Importance of right communication with healthcare providers and patients about the new levothyroxine formulation: an expert opinion from Asia Pacific Thyroid Advisory Board. Curr Med Res Opin. (2024) 40:1533–6. doi: 10.1080/03007995.2024.2378984, PMID: 39104288

[40] Matrone A Faranda A Latrofa F Gambale C Stefani Donati D Molinaro E . Thyroglobulin changes are highly dependent on TSH in low-risk DTC patients not treated with radioiodine. J Clin Endocrinol Metab. (2020) 105:dgaa297. doi: 10.1210/clinem/dgaa297, PMID: 32453405

[41] Zafon C Díez JJ Galofré JC Cooper DS . Nodular thyroid disease and thyroid cancer in the era of precision medicine. Eur Thyroid J. (2017) 6:65–74. doi: 10.1159/000457793, PMID: 28589087 PMC5422742

[42] White C Weinstein MC Fingeret AL Randolph GW Miyauchi A Ito Y . Is less more? A microsimulation model comparing cost-effectiveness of the revised American thyroid association’s 2015 to 2009 guidelines for the management of patients with thyroid nodules and differentiated thyroid cancer. Ann Surg. (2020) 271:765–73. doi: 10.1097/SLA.0000000000003074, PMID: 30339630

[43] Francke AL Smit MC de Veer AJ Mistiaen P . Factors influencing the implementation of clinical guidelines for health care professionals: a systematic meta-review. BMC Med Inform Decis Mak. (2008) :8:38. doi: 10.1186/1472-6947-8-38, PMID: 18789150 PMC2551591

[44] Ringel MD Sosa JA Baloch Z Bischoff L Bloom G Brent GA . 2025 American thyroid association management guidelines for adult patients with differentiated thyroid cancer. Thyroid. (2025) 35:841–985. doi: 10.1177/10507256251363120, PMID: 40844370 PMC13090833

