# Supplementary material for: Assessment of levothyroxine therapy adequacy in low-risk differentiated thyroid carcinoma: a multicenter cohort study
Source: Front Endocrinol (Lausanne). 2025 Dec 12;16:1652862. doi: 10.3389/fendo.2025.1652862 (PMC12740901; doi:10.3389/fendo.2025.1652862)
Supplement: Supplementary file 1 [file Table1.docx]

**Assessment of Levothyroxine Therapy Adequacy in Low-Risk Differentiated Thyroid Carcinoma: A Multicenter Cohort Study**

**SUPPLEMENTARY MATERIAL**

**Table S1**. Methods for measuring serum thyroglobulin by the investigators participating in the study, indicating the detection limit of each assay

| **Manufacturer** | **Instrument** | **Investigator (number, %)** | **Patients (number, %)** | **Detection limit (ng/ml)** |
| --- | --- | --- | --- | --- |
| Abbott | Alinity | 2 (11.8) | 84 (8.3) | 0.09 |
| Beckman | DXI | 2 (11.8) | 139 (13.7) | 0.10 |
| Diasorin | Liaison | 1 (5.9) | 40 (3.9) |  |
| Roche | Cobas | 5 (29.4) | 346 (34.1) | 0.04-0.10 |
|  | Elecsys | 2 (11.8) | 153 (15.1) | 0.01-0.04 |
| Siemens | Atellica | 3 (17.6) | 172 (16.9) | 0.05-0.10 |
|  | Immulite 2000 | 2 (11.8) | 82 (8.1) | 0.50 |

**Table S2**. Methods for measuring serum thyrotropin by the investigators participating in the study, indicating the reference interval of the assay used

|  |  |  |  |  |
| --- | --- | --- | --- | --- |
|  |  |  |  |  |
| **Manufacturer** | **Instrument** | **Investigator (number, %)** | **Patients (number, %)** | **Reference interval (mcU/ml)** |
| Abbott | Alinity | 3 (17.6) | 155 (15.3) | 0.35-4.94 |
| Beckman | DXI | 3 (17.6) | 257 (25.3) | 0.30-5.30 |
| Roche | Cobas | 2 (11.8) | 86 (8.5) | 0.25-5.00 |
|  | Elecsys | 2 (11.8) | 153 (15.1) | 0.3-4.2 |
| Siemens | Advia Centaur | 1 (5.9) | 61 (6.0) | 0.55-5.0 |
|  | Atellica | 6 (35.3) | 304 (29.9) | 0.55-4.78 |

**Table S3**. Classification of patients into serum thyroglobulin response groups according to initial treatment modality and thyroglobulin quantification instrument

|  | **Serum thyroglobulin levels (ng/ml)** | |
| --- | --- | --- |
|  | **Immulite 2000** | **Other instruments** |
| **Total thyroidectomy and RAI ablation** |  |  |
| Excellent (Tg EX) | <0.50 | <0.20 |
| Indeterminate (Tg IN) | 0.50-0.99 | 0.20-0.99 |
| Biochemical incomplete (TG BI) | ≥1.00 | ≥1.00 |
|  |  |  |
| **Total thyroidectomy without RAI ablation** |  |  |
| Excellent (Tg EX) | <0.50 | <0.20 |
| Indeterminate (Tg IN) | 0.50-4.99 | 0.20-4.99 |
| Biochemical incomplete (TG BI) | ≥5.00 | ≥5.00 |
|  |  |  |
| **Lobectomy** |  |  |
| Excellent (Tg EX) | <30.00 | <30.00 |
| Biochemical incomplete (TG BI) | ≥30.00 | ≥30.00 |
|  |  |  |

**Table S4.** Results of univariable and multivariable logistic regression models to study the influence of several covariates as potential predictors of adequacy of levothyroxine treatment in patients who achieved an excellent response at 12 months (n=633)

|  | **Univariable** | | | **Model 1** | | | **Model 2** | | |
| --- | --- | --- | --- | --- | --- | --- | --- | --- | --- |
|  | **OR** | **95% CI** | **P** | **OR** | **95% CI** | **P** | **OR** | **95% CI** | **P** |
| **Gender, male** | 1.64 | 1.01-2.51 | 0.022 | 1.64 | 1.07-2.51 | 0.023 | 1.59 | 1.01-2.50 | 0.045 |
| **Age, yr** | 1.00 | 0.99-1.02 | 0.888 | 1.00 | 0.99-1.02 | 0.744 | 0.99 | 0.98-1.01 | 0.677 |
| **Histology, follicular** | 1.14 | 0.62-2.09 | 0.675 |  |  |  | 1.39 | 0.71-2.74 | 0.342 |
| **Tumor size, cm** | 0.89 | 0.76-1.03 | 0.114 |  |  |  | 0.96 | 0.81-1.14 | 0.652 |
| **Incidental** | 1.60 | 1.06-2.42 | 0.025 |  |  |  | 1.28 | 0.81-2.02 | 0.290 |
| **Total thyroidectomy** | 0.50 | 0.27-0.92 | 0.025 |  |  |  | 0.88 | 0.44-1.76 | 0.717 |
| **Radioiodine** | 0.41 | 0.28-0.59 | <0.001 |  |  |  | 0.44 | 0.28-0.68 | <0.001 |
| **Hypoparathyroidism** | 2.08 | 1.13-3.83 | 0.019 |  |  |  | 2.05 | 1.08-3.89 | 0.028 |

Abbreviations: OR odds ratio, CI confidence interval.

Model 1: demographic features (gender, age); model 2: in addition to the above, clinical, pathological and therapeutic variables.

**Table S5.** Results of univariable and multivariable logistic regression models to study the influence of several covariates as potential predictors of adequacy of levothyroxine treatment in patients who achieved an excellent response at last visit (n=761)

|  | **Univariable** | | | **Model 1** | | | **Model 2** | | |
| --- | --- | --- | --- | --- | --- | --- | --- | --- | --- |
|  | **OR** | **95% CI** | **P** | **OR** | **95% CI** | **P** | **OR** | **95% CI** | **P** |
| **Gender, male** | 1.33 | 0.92-1.91 | 0.126 | 1.31 | 1.91-1.89 | 0.143 | 1.31 | 0.85-2.01 | 0.219 |
| **Age, yr** | 0.99 | 0.99-1.01 | 0.856 | 1.00 | 0.99-1.01 | 0.715 | 1.00 | 0.98-1.01 | 0.961 |
| **Time of follow-up, mo** | 1.00 | 1.00-1.01 | 0.018 | 1.00 | 1.00-1.01 | 0.019 | 1.00 | 0.98-1.00 | 0.650 |
| **Histology, follicular** | 1.37 | 0.84-2.23 | 0.203 |  |  |  | 1.23 | 0.68-2.20 | 0.494 |
| **Tumor size, cm** | 1.08 | 0.96-1.21 | 0.189 |  |  |  | 1.04 | 0.89-1.21 | 0.622 |
| **Incidental** | 1.17 | 0.83-1.65 | 0.671 |  |  |  | 1.21 | 0.79-1.86 | 0.370 |
| **Total thyroidectomy** | 0.78 | 0.44-1.40 | 0.782 |  |  |  | 0.87 | 0.40-1.91 | 0.730 |
| **Radioiodine** | 1.02 | 0.74-1.41 | 0.898 |  |  |  | 1.12 | 0.72-1.75 | 0.615 |
| **Hypoparathyroidism** | 0.92 | 0.54-1.57 | 0.758 |  |  |  | 0.94 | 0.51-1.75 | 0.855 |
| **Health problem** | 0.88 | 0.53-1.45 | 0.614 |  |  |  | 0.77 | 0.42-1.38 | 0.377 |
| **Dose instability*** | 0.15 | 0.10-0.22 | <0.001 |  |  |  | 0.14 | 0.09-0.21 | <0.001 |

Abbreviations: OR odds ratio, CI confidence interval.

Model 1: demographic features (gender, age) and time of follow-up; model 2: in addition to the above, clinical, pathological and therapeutic variables.

*n=706.

**Table S6.** Results of univariable and multivariable logistic regression models to study the influence of several covariates as potential predictors of adequacy of levothyroxine treatment in patients who achieved an indeterminate or biochemical incomplete response at 12 months (n=375)

|  | **Univariable** | | | **Model 1** | | | **Model 2** | | |
| --- | --- | --- | --- | --- | --- | --- | --- | --- | --- |
|  | **OR** | **95% CI** | **P** | **OR** | **95% CI** | **P** | **OR** | **95% CI** | **P** |
| **Gender, male** | 0.73 | 0.39-1.36 | 0.322 | 0.73 | 0.39-1.37 | 0.327 | 0.54 | 0.27-1.09 | 0.543 |
| **Age, yr** | 1.01 | 0.99-1.02 | 0.575 | 1.01 | 0.99-1.02 | 0.589 | 1.01 | 0.99-1.03 | 0.183 |
| **Histology, follicular** | 2.50 | 1.15-5.40 | 0.020 |  |  |  | 1.97 | 0.84-4.65 | 0.119 |
| **Tumor size, cm** | 1.27 | 1.05-1.54 | 0.016 |  |  |  | 1.14 | 0.89-1.47 | 0.297 |
| **Incidental** | 0.67 | 0.39-1.15 | 0.145 |  |  |  | 0.98 | 0.51-1.87 | 0.939 |
| **Total thyroidectomy** | 12.67 | 1.71-94.0 | 0.013 |  |  |  | 8.57 | 1.11-66.1 | 0.039 |
| **Radioiodine** | 2.33 | 1.41-3.84 | 0.001 |  |  |  | 1.68 | 0.94-3.00 | 0.079 |
| **Hypoparathyroidism** | 0.71 | 0.30-1.69 | 0.422 |  |  |  | 0.63 | 0.26-1.54 | 0.310 |

Abbreviations: OR odds ratio, CI confidence interval.

Model 1: demographic features (gender, age); model 2: in addition to the above, clinical, pathological and therapeutic variables.

**Table S7.** Results of univariable and multivariable logistic regression models to study the influence of several covariates as potential predictors of adequacy of levothyroxine treatment in patients who achieved an indeterminate or biochemical incomplete response at last visit (n=213)

|  | **Univariable** | | | **Model 1** | | | **Model 2** | | |
| --- | --- | --- | --- | --- | --- | --- | --- | --- | --- |
|  | **OR** | **95% CI** | **P** | **OR** | **95% CI** | **P** | **OR** | **95% CI** | **P** |
| **Gender, male** | 0.97 | 0.96-1.02 | 0.965 | 0.99 | 0.45-2.19 | 0.979 | 0.59 | 0.22-1.63 | 0.311 |
| **Age, yr0.99** | 0.99 | 0.97-1.02 | 0.532 | 0.97 | 0.97-1.02 | 0.463 | 0.98 | 0.96-1.01 | 0.241 |
| **Time of follow-up, mo** | 1.00 | 0.99-1.00 | 0.637 | 1.00 | 0.99-1.00 | 0.546 | 1.00 | 0.99-1.00 | 0.180 |
| **Histology, follicular** | 1.46 | 0.36-5.88 | 0.592 |  |  |  | 1.13 | 0.21-6.23 | 0.887 |
| **Tumor size, cm** | 1.36 | 1.05-1.77 | 0.018 |  |  |  | 1.42 | 0.99-2.03 | 0.055 |
| **Incidental** | 0.59 | 0.28-1.21 | 0.150 |  |  |  | 0.93 | 0.37-2.34 | 0.885 |
| **Total thyroidectomy** | 1.74 | 0.57-5.33 | 0.330 |  |  |  | 0.87 | 0.24-3.18 | 0.841 |
| **Radioiodine** | 1.43 | 0.75-2.71 | 0.279 |  |  |  | 0.97 | 0.41-2.30 | 0.943 |
| **Hypoparathyroidism** | 0.82 | 0.26-2.59 | 0.738 |  |  |  | 1.44 | 0.39-5.33 | 0.587 |
| **Health problem** | 1.35 | 0.45-4.03 | 0.597 |  |  |  | 1.64 | 0.47-5.68 | 0.435 |
| **Dose instability*** | 0.60 | 0.30-1.21 | 0.154 |  |  |  | 0.45 | 0.21-0.99 | 0.046 |

Abbreviations: OR odds ratio, CI confidence interval.

Model 1: demographic features (gender, age) and time of follow-up; model 2: in addition to the above, clinical, pathological and therapeutic variables.

*n=188.
